# Heregulin-1ß and HER3 in hepatocellular carcinoma: status and regulation by insulin

**DOI:** 10.1186/s13046-016-0402-3

**Published:** 2016-08-11

**Authors:** Corina Buta, Eva Benabou, Marie Lequoy, Hélène Régnault, Dominique Wendum, Fatiha Meratbene, Hamza Chettouh, Lynda Aoudjehane, Filomena Conti, Yves Chrétien, Olivier Scatton, Olivier Rosmorduc, Françoise Praz, Laetitia Fartoux, Christèle Desbois-Mouthon

**Affiliations:** 1 Sorbonne Universités, UPMC Univ Paris 06, INSERM, Saint-Antoine Research Center, 34 rue Crozatier, F-75012 Paris, France; 2 Department of Hepatology, AP-HP, Saint-Antoine Hospital, F-75012 Paris, France; 3 Department of Hepatology, AP-HP, Pitié-Salpétrière Hospital, F-75013 Paris, France; 4 Department of Pathology, AP-HP, Saint-Antoine Hospital, F-75012 Paris, France; 5 Histomorphology Platform, UMS 30 Lumic, F-75012 Paris, France; 6 Human HepCell, Saint-Antoine Hospital, F-75012 Paris, France; 7 Department of Hepatobiliary Surgery and Liver Transplantation, AP-HP, Pitié-Salpétrière Hospital, F-75013 Paris, France

**Keywords:** HER3/ERBB3, Insulin receptor, Hepatitis virus, Liver cancer

## Abstract

**Background:**

The heregulin-1ß/HER3-driven pathway is implicated in several epithelial malignancies and its blockade is currently undergoing clinical investigation. Paradoxically, the status and the regulation of this pathway is poorly known in hepatocellular carcinoma (HCC).

**Methods:**

Using 85 HCC obtained after tumour resection, heregulin-1ß and HER3 expression was evaluated by real-time RT-PCR, ELISA and/or immunohistochemistry. Statistics were performed to analyze associations between gene expression and clinicopathological parameters. The effects of insulin on the heregulin-1ß/HER3 pathway was investigated in four HCC cell lines.

**Results:**

HER3 mRNA was upregulated in 52 % of tumours, while heregulin-1ß mRNA was downregulated in 82 %. Hepatitis B and C viral infections were respectively associated with high and low HER3 mRNA expression. No association was seen between neither HER3 or heregulin-1ß mRNA and prognostic factors, survival or recurrence. Immunohistochemistry showed predominant cytoplasmic staining of HER3 in tumours but the staining was nonreproducible. HER3 mRNA and protein levels were not correlated in liver tissues. In HCC cells, insulin promoted HER3 proteasomal degradation and inhibited heregulin-1ß stimulation of cell migration. HER3 and insulin receptor co-immunoprecipitated in these cells. The loss of insulin receptor expression by RNA interference sensitized cells to heregulin-1ß-induced AKT phosphorylation.

**Conclusions:**

Autocrine heregulin-1ß loop is uncommon in HCC and HER3 mRNA expression is differentially influenced by hepatitis viruses. Insulin is a negative regulator of HER3 protein expression and function in HCC cells. Altogether these data may explain why HER3 and heregulin-1ß expression have no prognostic value and suggest that HCC patients are unlikely to derive benefit from HER3-targeted monotherapies.

**Electronic supplementary material:**

The online version of this article (doi:10.1186/s13046-016-0402-3) contains supplementary material, which is available to authorized users.

## Background

Hepatocellular carcinoma (HCC) is a primary tumour of the liver whose incidence has steadily increased in recent years, reaching the fifth place worldwide. HCC has a dismal prognosis and it ranks second in terms of mortality. A minority of patients benefit from curative therapies (liver transplantation, tumour resection) and a high incidence of postoperative recurrence is observed after resection. Tumour recurrence is the major cause of death following resection [1–3]. In this context, efforts must be pursued to better characterize HCC at genetic, molecular and cellular levels to identify key oncogenic pathways and therapeutical targets.

HER3 (ErbB3) belongs to the HER family including HER1 (ErbB1 or epidermal growth factor receptor (EGFR)), HER2 (ErbB2), and HER4 (ErbB4). Heregulin-1ß (or neuregulin-1ß) is a high affinity ligand for HER3. HER3 has minimal tyrosine kinase activity and its full activation upon heregulin-1ß binding, depends on its association with other HER members such as EGFR and HER2. Activated HER3 has six tyrosine-containing binding sites for the p85 regulatory subunit of PI3K in the cytoplasmic tail, making HER3 a major regulator of AKT-dependent signalling [4–6]. These last years, the heregulin-1ß/HER3 signalling axis has generated much interest in medical oncology. Indeed, high tumour expression of HER3 has been shown to be predictive of tumour progression and poor survival in patients with ovarian [7], breast [8, 9], melanoma [10] or gastric [11, 12] cancers. The presence of paracrine/autocrine heregulin-1ß loops also defines a subset of agressive tumours with higher recurrence in head and neck squamous cell carcinomas [13, 14]. Yet, the poor prognostic value of HER3 and/or heregulin-1ß remains controversial in other cancers such as bladder cancer [15], uveal melanoma [16] and lung adenocarcinoma [17].

Data regarding the HER3 status in HCC are scarce. Available data have been essentially obtained from populations of Asian patients with viral hepatitis. A Japanese study reported that 64 out of 84 HCC were positive for cytoplasmic HER3 by immunohistochemistry [18]. The transcriptomic profile of 37 hepatitis B virus (HBV)-related HCC showed that HER3 mRNA was one of the most frequently induced [19]. More recently, a Taiwanese group reported that upregulation of HER3 mRNA was associated with HBV etiology, microvascular invasion, early recurrence and poor clinical outcome in 71 patients with HCC [20]. No data are available regarding heregulin-1ß expression in HCC.

The present study was defined to gain information regarding the status of HER3 and heregulin-1ß in a French collection of HCC. We observed that HER3 mRNA expression was increased in 52 % of 85 tumours while heregulin-1ß mRNA expression was reduced in 82 %. No prognostic value was found for HER3 or heregulin-1ß mRNA expression in this collection. In addition, no correlation was observed between HER3 mRNA and protein levels. The analysis of the post-transcriptional regulation of HER3 in HCC cell lines revealed that the heregulin-1ß/HER3 signalling pathway was controlled negatively by insulin at different levels.

## Methods

### Patients and liver tissue specimens

Eighty-five HCC (T) and paired adjacent non-tumour (NT) liver tissues were collected from patients undergoing curative liver resection for HCC at the Saint-Antoine hospital (Paris, France). Clinicopathological characteristics are summarized in Table 1. Part of this collection was used in our previous study where it was designated as collection #2 [21]. All patients gave informed consent to the study, which was conducted in accordance with the French laws and regulations (CNIL n° 1913901 v 0).

**Table 1:** Clinicopathological characteristics of 85 patients with HCC

Age at surgery (years)	
Median [range]	64.0 [18–85]
Sex ratio (M/F)	5.1 (71/14)
Etiology of chronic liver disease, *n* (%)	
HCV infection HBV infection Alcohol abuse Hemochromatosis NASH Combined viral hepatitis and alcohol Combined metabolic syndrome and alcohol Undetermined	21 (24.7) 27 (31.8) 5 (5.9) 2 (2.3) 11 (12.9) 5 (5.9) 8 (9.4) 11 (12.9)
Advanced fibrosis/cirrhosis, *n* (%)	49 (57.6)
Maximal tumour size, mean ± SD (mm)	65.2 ± 38.5
AFP (≥400 ng/ml), *n* (%)^a^	15 (17.6)
Multiplicity, *n* (%)	18 (21.2)
Tumour grade	
Well differentiated, *n* (%) Moderately differentiated, *n* (%) Poorly differentiated, *n* (%)	21 (24.7) 41 (48.2) 23 (27.0)
CK19 expression, *n* (%)^b^	17 (20.0)
Microvascular invasion, *n* (%)	40 (47.0)
Satellite nodules, *n* (%)	26 (30.5)
Recurrence, *n* (%)	45 (52.9)
Recurrence before 2 years, *n* (%)	40 (47.1)
Delay to recurrence (months), median [range]	7.8 [1–61]
Overall mortality, *n* (%)	29 (34.1)

*AFP* α-fetoprotein, *CK19* cytokeratin 19, *HCV* hepatitis C virus

*HBV* hepatitis B virus, *NASH* non alcoholic steatohepatitis

^a^four missing data

^b^three missing data

### Cell culture and treatments

HepG2, Hep3B, and Huh7 cells were obtained from the American Type Culture Collection (ATCC). PLC/PRF5 cells were provided by Dr Christine Perret (Institut Cochin, France). Cell line authentication was routinely performed by using a panel of nine ATCC short tandem repeats. Cell lines were cultured as previously reported [22] and routinely controlled for mycoplasma contamination. Primary cultures of human hepatocytes were established as previously described [23]. In some experiments, serum-deprived cells were treated with insulin, IGF-II (Sigma-Aldrich), heregulin-1ß (R&D Systems), cycloheximide, bafilomycin A1 (Sigma-Aldrich) and/or MG-132 (Cell Signaling Technology).

### Western blotting and ELISA

Protein electrophoresis and transfert to nitrocellulose were performed according to standard procedures. The primary antibodies are summarized in Additional file 1: Table S1. Blot revelations and quantifications were performed using ChemiDoc™ Touch Imaging System (BIORAD). Total amounts of HER3 and heregulin-1β were quantified in human liver tissue extracts by ELISA according to manufacturer’s instructions (R&D Systems).

### RNA interference

The expression of specific mRNA was downregulated using a mixture of four siRNAs (100 nmol/L, ON-TARGET*plus* SMARTpool, Dharmacon) and Dharmafect 4 (Dharmacon). Controls were performed using a non-targeting siRNA pool (Dharmacon).

### RNA isolation and analysis of gene expression

Total RNA was extracted from cell cultures using RNeasy Mini kit (Qiagen). For liver tissues, a preliminary RNA extraction step was performed using TRIzol Reagent (Life Technologies). Quantitative measurements of transcripts were performed by real-time PCR on a LightCycler 480 instrument (Roche) using SYBR Green chemistry and specific primers (Additional file 1: Table S2). For each sample, gene expression was normalized to that of hypoxanthine guanine phosphoribosyltransferase mRNA content and was expressed relatively to the same calibrator. The relative quantity of each target gene was determined from replicate samples using the formula 2^-∆∆Ct^.

### Immunohistochemistry

Paraffin-embedded 4-μm sections were dewaxed in xylene and rehydrated in graded alcohol series and microwave antigen retrieval was performed in 10 mM citrate buffer pH 6 during 15 min. Primary antibody detection was performed using Novolink Polymer Detection System (Novocastra, Leica Biosystems) according to the manufacturer’s protocol on an automated staining system (Dakocytomation®). Aminoethyl carbazole was used to reveal the peroxidase activity. The sections were counterstained with haematoxylin. Four different HER3 monoclonal antibodies were tested (Additional file 1: Table S1) but only the clone RTJ1 (Thermo Scientific, 1:30 dilution, overnight at 4 °C) gave signals. The clone RTJ1 was validated by siRNA-mediated knockdown and Western blotting (Additional file 2: Figure S1).

### Immunofluorescence analysis

Cells seeded on glass coverslips were fixed with 4 % paraformaldehyde, blocked with 1 % BSA and 10 % goat serum in PBS, followed by overnight incubation with a 1:250 dilution of the primary antibody in PBS at 4 °C (Additional file 1: Table S1). Cells were then incubated with a 1:200 dilution of conjugated secondary antibody (Alexa Fluor® 488 or 546 dye) in PBS for 1 h at room temperature. The slides were counterstained with 4′,6-diamidino-2-phenylindole (DAPI) for nuclei detection. Fluorescence was visualized using an immunofluorescence microscope (Leica Microsystems) with a DFC300 FX digital camera.

### Cell migration assays

Migration was performed in 6.5 mm Transwell® with 8-μm pore polycarbonate membrane insert (Corning). Cells (1 × 10^5^) in medium without serum were plated in the upper chamber and medium containing 10 % fetal bovine serum (FBS) was added in the lower chamber as a chemoattractant. After 24 h, cells were treated with or without insulin or heregulin-1β for 24 h. Cells that had not migrated were removed from the upper surface of inserts by using cotton-tipped swabs and migrated cells that were attached to the lower surface were enumerated by microscopy following fixation by 4 % paraformaldehyde for 15 min and nucleic acid staining with DAPI. Four random fields were counted per insert. For the wound-healing assay, cells (5 × 10^4^) were seeded in a 24-well dish, incubated for 24 h in complete medium and serum-harvested overnight. The cell monolayers were pretreated with mitomycin C (1 μg/ml) for 2 h to inhibit cell proliferation, scraped with a p200 pipet tip and washed. Cells were then treated with or without insulin or heregulin-1β. Photographs were taken at 0 and 48 h with a phase-contrast microscope and at least ten fields were recorded for each treatment.

#### Statistical analysis

Comparison of mRNA expression between tumours and adjacent liver tissue was performed using Wilcoxon signed-rank test. The association between gene expression (T/NT ratio) and clinicopathological features was evaluated using the Mann–Whitney U-test. Survival analysis was done by the Kaplan–Meier method and the groups were compared with the log-rank test. Data from *in vitro* experiments were reported as means +/− SEM of at least three independent experiments. The comparisons of different groups were carried out using Mann–Whitney test. Differences were considered statistically significant at *p* < 0.05.

## Results

### HER3 and heregulin-1ß mRNA expression is not associated with clinicopathological markers of tumour progression and reduced survival in HCC

**Fig. 1:**
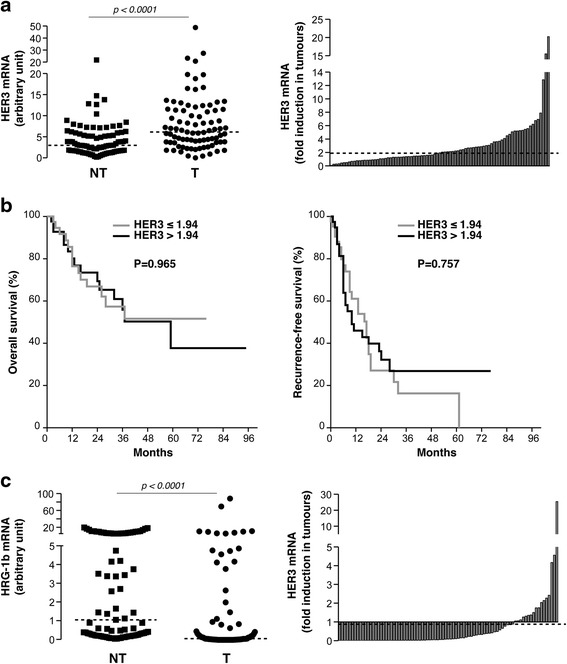
HER3 and heregulin-1ß mRNA expression in HCC. **a** HER3 mRNA expression was evaluated by RT-qPCR in 85 human paired T/NT liver tissue samples (*left*). The distribution of HER3 T/NT ratio is presented on the right side. **b** Kaplan-Meier analysis of the probabilities of overall survival (*left*) and recurrence-free survival (*right*) according to the upregulation of HER3 mRNA. **c** Heregulin-1ß mRNA expression was evaluated by RT-qPCR in 85 human paired T/NT liver tissue samples (*left*). The distribution of heregulin-1ß T/NT ratio is presented on the right side

We analysed the expression of HER3 mRNA in a French collection of 85 resected HCC. We observed that HER3 mRNA was increased in 52 % of HCC (T) *versus* paired non-tumour (NT) liver tissue with a median fold-induction of 1.94 (Fig. 1a). High HER3 T/NT ratios were associated with HBV, while lower values were associated with HCV (Table 2). Except for tumour size, the HER3 T/NT ratio was not associated with histological and biological markers reminiscent of tumours with poor outcome such as CK19 expression, satellite nodules, multiplicity, microvascular invasion and high serum levels of AFP (Table 2). The HER3 T/NT ratio was higher in well/moderately differentiated tumours than in poorly differentiated tumours. Finally, there was no difference in overall survival (OS) or recurrence-free survival (RFS) in patients with high or low HER3 T/NT ratio (Fig. 1b).

**Table 2:** Relations between HER3 and heregulin-1ß mRNA fold inductions (T/NT) and the pathological characteristics of 85 HCC

	Number	HER3 mRNA fold induction (T/NT)^a^	*P* values	Heregulin-1ß fold induction (T/NT)^a^	*P* values
HBV					
Yes No	27 58	2.38 [0.29–20.25] 1.55 [0.07–15.51]	0.033	0.02 [0.00–4.56] 0.13 [0.00–25.46]	0.075
HCV					
Yes No	21 64	1.30 [0.25–12.86] 2.21 [0.07–20.25]	0.016	0.06 [0.00–4.17] 0.095 [0.00–25.46]	0.724
NASH					
Yes No	11 74	2.74 [0.69– 5.30] 1.60 [0.07–20.25]	0.313	0.33 [0.02–2.04] 0.05 [0.00–25.46]	0.074
MS + alcohol					
Yes No	8 77	1.32 [0.85–4.50] 2.06 [0.07–20.25]	0.425	0.715 [0.05–25.46] 0.06 [0.00–4.56]	0.011
Alcohol					
Yes No	5 80	2.11 [0.96–6.77] 1.85 [0.07–20.25]	0.727	0.10 [0.00–0.89] 0.08 [0.00–25.46]	0.783
Advanced fibrosis/cirrhosis					
Yes No	50 35	1.52 [0.25–20.25] 2.60 [0.07–15.51]	0.150	0.090 [0.00–4.17] 0.090 [0.00–25.46]	0.719
AFP^b^					
< 400 ng/ml ≥ 400 ng/ml	47 34	1.85 [0.07–20.25] 2.09 [0.25–15.51]	0.867	0.13 [0.00–4.56] 0.05 [0.00–25.46]	0.293
Tumour size					
< 5 cm ≥ 5 cm	44 41	1.47 [0.25–7.89] 2.68 [0.07–20.25]	0.019	0.09 [0.00–4.56] 0.09 [0.00–25.46]	0.631
Multiplicity					
Yes No	18 67	2.05 [0.32–20.25] 1.94 [0.07–7.89]	0.796	0.24 [0.00–4.17] 0.06 [0.00–25.46]	0.310
Satellite nodules					
Yes No	26 59	2.11 [0.07–20.25] 1.58 [0.25–7.89]	0.458	0.15 [0.00–25.46] 0.05 [0.00–4.56]	0.164
Differentiation					
Well/moderate Poor	61 24	2.22 [0.29–20.25] 1.43 [0.07–6.77]	0.021	0.23 [0.00–25.46] 0.01 [0.00–1.76]	0.001
CK19 expression^c^					
< 5 % ≥ 5 %	65 17	1.85 [0.25–20.25] 2.44 [0.07–15.51]	0.926	0.11 [0.00–25.46] 0.01 [0.00–1.06]	0.006
Microvascular invasion					
Yes No	41 44	2.08 [0.07–20.25] 1.56 [0.29–6.94]	0.194	0.08 [0.00–25.46] 0.09 [0.00–4.56]	0.624

*AFP* α-fetoprotein, *CK19* cytokeratin 19, *HCV* hepatitis C virus

*HBV* hepatitis B virus, *MS* metabolic syndrome, *NASH* non alcoholic steatohepatitis

^a^Values are expressed as median [range]

^b^four missing data

^c^three missing data

All statistical analyses were performed using Mann-Whitney test

Similar analyses were conducted for heregulin-1ß ligand. Heregulin-1ß mRNA levels were lower in 82 % of HCC compared with surrounding liver tissue (median fold-ratio of 0.09) (Fig. 1c). The heregulin-1ß expression ratio (T/NT) correlated neither with tumour size, multiplicity, microvascular invasion, OS nor RFS (Table 2 and Additional file 3: Figure S2); it was higher in CK19-negative and well/moderately differentiated tumours.

### HER3 mRNA and protein levels are not correlated in HCC and adjacent liver tissue

**Fig. 2:**
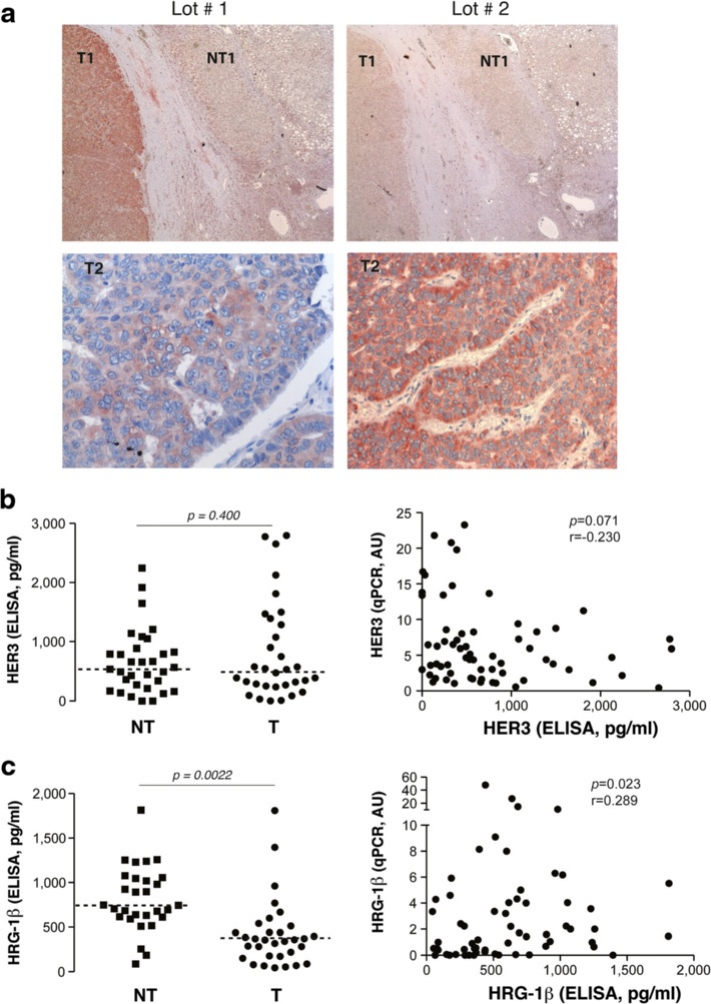
HER3 protein expression in HCC. **a** HER3 detection by immunohistochemistry with RTJ1 antibody revealed cytoplasmic staining in HCC. Representative pictures of two tumours (T1, T2) are shown. HER3 staining was non reproducible in terms of intensity between two lots of antibody (compare *left* and *right panels*). **b** *left*, HER3 protein expression evaluated by ELISA in 32 paired T/NT liver tissue samples; *right*, correlations between HER3 mRNA and protein levels. **c** *left*, heregulin-1ß (HRG-1ß) protein expression evaluated by ELISA in 32 paired T/NT liver tissue samples; *right*, correlations between heregulin-1ß mRNA and protein levels

We then attempted to investigate HER3 protein expression by immunohistochemistry. Out of four primary antibodies tested, only the RTJ1 clone yielded staining. This staining was exclusively cytoplasmic. This finding raises questions regarding the validity of these signals since HER3 is supposed to be also localized at cell membrane. Moreover, HER3 staining turned out to be nonreproducible in terms of intensity as illustrated in Fig. 2a and no reliable association studies could be performed from these analyses.

Thirty-two paired T/NT tissue samples previously analysed for HER3 mRNA expression were also measured for HER3 protein levels by ELISA. We observed no statistical difference between HER3 protein contents in tumours *vs* nontumour liver tissues (Fig. 2b, left). Spearman correlation analysis indicated no correlation between HER3 mRNA levels (evaluated by real-time PCR) and HER3 protein levels (evaluated by ELISA) (Spearman *r* = −0.230; *p* = 0.071; Fig. 2b, right). In contrast, heregulin-1ß protein levels were decreased in tumours compared with nontumour liver tissues (Fig. 2c, left) and heregulin-1ß mRNA and protein levels tended to be positively correlated (Spearman *r* = 0.289; *p* = 0.023; Fig. 2c, right). The lack of correlation between HER3 transcript and protein levels suggested that HER3 is subjected to post-transcriptional and/or post-translational regulation in human liver samples.

### Insulin promotes HER3 degradation in human hepatocytes and HCC cell lines

**Fig. 3:**
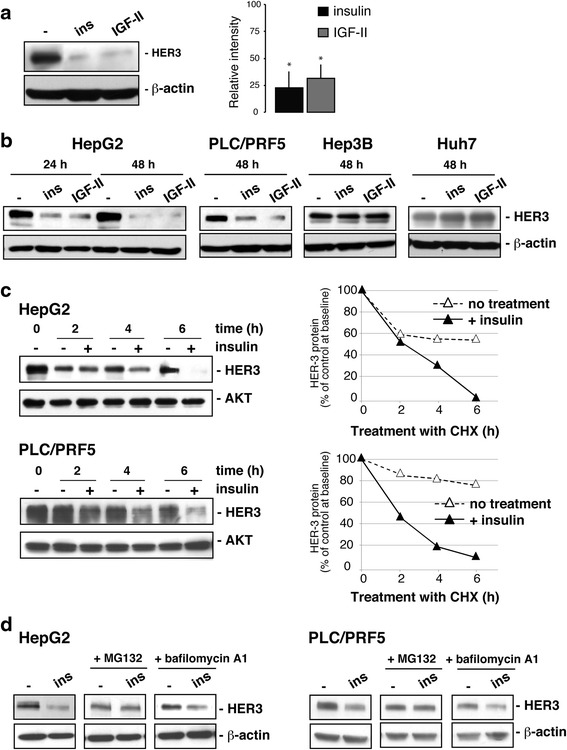
Effects of insulin on HER3 protein in normal hepatocytes and HCC cell lines. **a**–**b** Human normal hepatocytes and liver cancer cell lines (HepG2, PLC/PRF5, Hep3B, Huh7) were treated with 10^−8^ M insulin (ins) or IGF-II for 24 and 48 h. Whole-cell lysates (20 ug) were analysed by Western blot for HER3 expression. ß-actin detection was performed to control protein loading. **c** Serum-starved HepG2 and PLC/PRF5 cells were treated for 24 h with or without 10^−8^ M insulin, then with cycloheximide (CHX, 40 ng/ml) for different durations, analyzed by Western blot for HER3 expression and quantified. AKT detection was performed to control protein loading. **d** Serum-starved HepG2 and PLC/PRF5 cells were treated for 24 h with or without 10^−8^ M insulin, 10 uM MG132, or 100 nM bafilomycin and analyzed by Western blot for HER3 expression. ß-actin detection was performed to control protein loading. Blots are representative of three independent experiments

Limited data are available regarding the post-transcriptional and post-translational mechanisms controlling HER3 expression in hepatocytes. A few years ago, Carver and colleagues reported that in cultured rat hepatocytes insulin impaired heregulin-1ß signalling by promoting HER3 downregulation [24]. We examined whether such a mechanism was relevant to human normal and transformed hepatocytes, that expressed both HER3 and IR receptors [21, 22]. As shown in Fig. 3a, insulin down-regulated HER3 protein expression in primary cultures of normal human hepatocytes. A similar effect was observed in the presence of IGF-II, another ligand to the insulin receptor (IR). Insulin and IGF-II also decreased HER3 protein levels in HepG2 and PLC/PRF5 cancer cells but not in Hep3B and Huh7 cells (Fig. 3b). There was no decrease in HER3 mRNA levels after insulin treatment in HepG2 and PLC/PRF5 cells (Additional file 4: Figure S3A), showing that the effect of insulin was posttranscriptional. To determine whether the decreased levels of HER3 in insulin-stimulated HCC cells were due to increased degradation, we blocked the *de novo* protein synthesis with cycloheximide and analyzed the rates of HER3 decay. The half-life of HER3 in insulin-stimulated cells was shorter (2–3 h) than in unstimulated cells (>6 h) (Fig. 3c) indicating that insulin destabilizes HER3 protein. We then examined how insulin promoted HER3 degradation. First, we observed that treatment of HepG2 and PLC/PRF5 cells with the specific proteasome inhibitor MG132 for 24 h reversed the effect of insulin on HER3 downregulation while bafilomycin A, a selective inhibitor of lysosomal v-ATPase, was ineffective (Fig. 3d). This indicated that the negative regulation of HER3 protein levels by insulin was a proteasome-mediated process. Additionally, it seemed that the effect of insulin did not require the involvement of NRDP1, an E3 ubiquitin ligase, which can control the steady-state levels of HER3 [25]. Indeed, insulin did not modify NRDP1 expression and more importantly siRNA-induced downregulation of NRDP1 expression did not impact insulin effect on HER3 expression (Additional file 4: Figure S3 B and C).

### Insulin inhibits heregulin-1ß stimulation of migration in HCC cell lines

**Fig. 4:**
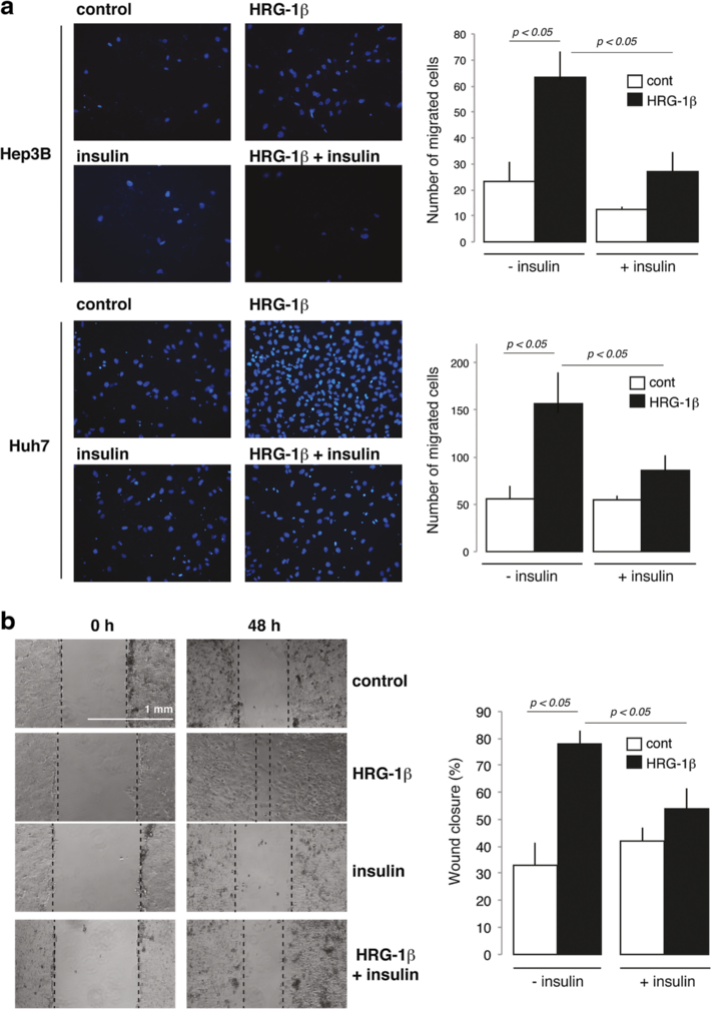
Effects of insulin on heregulin-1ß stimulation of cell migration in HCC cell lines. **a** Hep3B and Huh7 cells seeded on Transwell® inserts were treated for 24 h with 10^−8^ M insulin and/or 50 ng/ml heregulin-1ß (HRG-1ß). Migrated cells were enumerated by microscopy following nucleic acid staining with DAPI. **b** Huh7 cells were submitted to a wound-healing assay by scraping cell layer with a p200 pipet tip and were treated for 48 h with 10^−8^ M insulin and/or 50 ng/ml heregulin-1ß. The wound closure (% of H0) was measured

We then examined whether insulin impacted heregulin-1ß/HER3-dependent biological effects in Hep3B and Huh7 cell lines in which the hormone did not down-regulate HER3 expression. As shown in Fig. 4a, heregulin-1ß promoted cell migration as evaluated by Transwell migration assays in these cell lines while it was ineffective to stimulate proliferation and viability (Additional file 5: Figure S4). Insulin was a potent mitogen in these cell lines [21] but had no effect on cell migration (Fig. 4a). When insulin was combined to heregulin-1ß, we observed that the promigratory effect of heregulin-1ß was reduced in Hep3B and Huh7 cell lines (Fig. 4a). The ability of insulin to counteract heregulin-1ß migratory effect was confirmed in Huh7 cells using a wound healing assay (Fig. 4b).

### HER3 is associated to IR in HCC cell lines

**Fig. 5:**
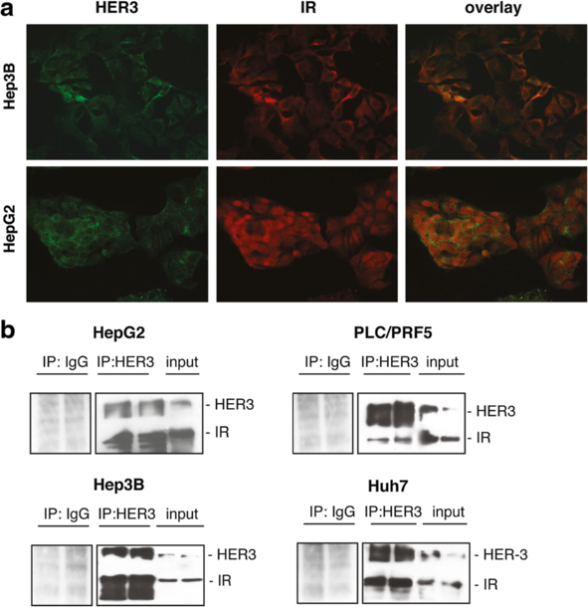
Association of HER3 to IR in HCC cells. **a** Hep3B and HepG2 cells were analyzed for HER3 and IR expression by immunofluorescence. **b** Whole-cell lysates from HCC cells were immunoprecipitated with a HER3 antibody and analyzed by Western blot using an anti-IR antibody. In parallel, negative controls were performed by immunoprecipitating the same lysates with a rabbit IgG. Pictures and blots are representative of two independent experiments

As a first step to investigate the mechanisms underlying the functional interaction between heregulin-1ß- and insulin-dependent pathways, we examined the cellular localization of HER3 and IR by immunofluorescence (Fig. 5a). In HCC cell lines, the two receptors showed a partially overlapping pattern of distribution. A potential interaction between IR and HER3 in HCC cells was next assessed by immunoprecipitation. HER3 was immunoprecipitated from whole-cell lysates, and the resulting precipitates were subjected to immunoblot analysis with an antibody to IR. In all cell lines, we observed that HER3 coimmunoprecipitated with IR (Fig. 5b).

### Insulin inhibits the heregulin-1ß/HER3/AKT pathway in HCC cell lines

**Fig. 6:**
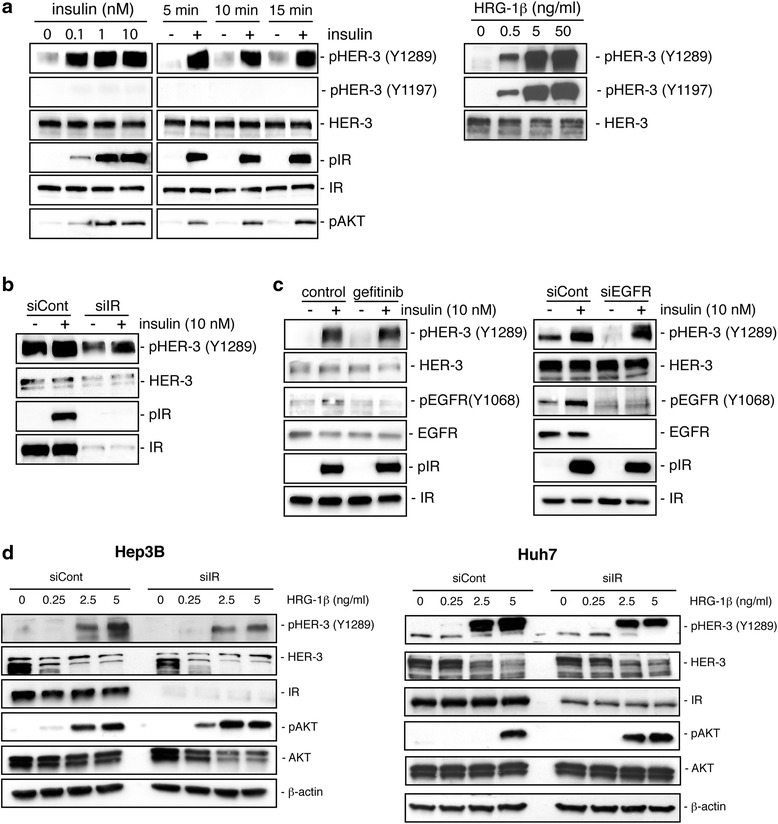
Effects of insulin on heregulin-1ß/HER3 signalling in HCC cell lines. **a** Hep3B cells were treated with insulin (*left:* increasing doses during 10 min; *right:* 10^−8^ M for different times) or with heregulin-1ß (HRG-1ß increasing doses during 10 min). Whole-cell lysates (20 µg) were analysed by Western blot for phosphorylation and expression of HER3, IR and/or AKT. **b** Hep3B cells were transiently transfected with a control (siCont) or siRNA directed against IR (siIR) and then treated with or without 10^−8^ M insulin for 10 min. Whole-cell lysates (20 μg) were analysed by Western blot for phosphorylation and expression of HER3 and/or IR. **c** Hep3B cells were treated or not with 2 uM gefitinib (*left*) or transiently transfected with control (siCont) or siRNA directed against EGFR (siEGFR) (*right*) and then treated with or without 10^−8^ M insulin for 10 min. Whole-cell lysates (20 µg) were analysed by Western blot for phosphorylation and expression of HER3, EGFR and/or IR. **d** Hep3B (*left*) and Huh7 (*right*) cells were transiently transfected with a control (siCont) or siRNA directed against IR (siIR) an then treated with or without increasing doses of heregulin-1ß for 10 min. Whole-cell lysates (20 µg) were analysed by Western blot for phosphorylation and expression of HER3, AKT and/or IR

We then examined the impact of insulin on the heregulin-1ß/HER3 signalling axis in HCC cells. The effect of insulin on HER3 phosphorylation was evaluated by Western blot by analysing two major tyrosine phosphorylation sites, Y1289 and Y1197. Strikingly, the hormone induced an increase of HER3 Y1289 phosphorylation in Hep3B cells while it was ineffective to promote HER3 Y1197 phosphorylation (Fig. 6a, *left*). As a comparison, both tyrosine residues were potently phosphorylated by heregulin-1ß (Fig. 6a, *right*). Insulin-induced phosphorylation of HER3 Y1289 was reduced when IR expression was downregulated with siRNA (Fig. 6b). The stimulatory effect of insulin on HER3 Y1289 phosphorylation was observed in the three other cell lines but to a lesser extent (Additional file 6: Figure S5). As insulin also promoted EGFR phosphorylation in HCC cells (Fig. 6c), we wondered whether insulin induction of Y1289 HER3 phosphorylation required EGFR activity. The effect of insulin on HER3 was maintained in the presence of gefitinib, an EGFR TKI, or after EGFR downregulation with siRNA suggesting that it occurred independently of EGFR activation (Fig. 6c). Finally, we examined the consequence of IR downregulation with siRNA on HER3 and AKT phosphorylation in the presence of increasing doses of heregulin-1ß. As shown in Fig. 6d, HER3 phosphorylation tended to be reduced while AKT phosphorylation was enhanced and detectable at lower doses of ligand after IR depletion. Altogether, these data indicate that HER3 is in a close proximity to IR in HCC cells and that IR exerts a negative constraint on heregulin-1ßlHER3 that could involve IR-mediated HER3 Y1289 phosphorylation.

## Discussion

Our study shows that HER3 mRNA is upregulated (52 %) in a French collection of 85 HCC compared with adjacent nontumour tissue. In accordance with studies performed on Asian collections of HCC [19, 20], we observed that the upregulation of HER3 mRNA was associated to chronic HBV infection. Therefore, it is tempting to speculate that HBV may favour higher HER3 expression during liver carcinogenesis. In this setting, a recent *in vitro* study showed that the viral protein Hbx transcriptionally up-regulates HER3 expression in HCC cells [26]. Moreover, secreted HER3 has been shown to be a biomarker for early HCC in patients with chronic B hepatitis and cirrhosis [27]. By contrast, it has been recently demonstrated that HCV down-regulates HER3 expression at both transcript and protein levels in the Huh7 cell line [28]. Accordingly, we observed that HER3 mRNA levels were low in HCV-related HCC. Altogether, these data suggest that the expression of HER3 mRNA is regulated differentially by the viral factors contributing to HCC.

Fold induction for HER3 mRNA expression was also higher in well/moderately differentiated tumours than in poorly differentiated ones. In the same setting, studies conducted on a large panel of hepatoma cell lines showed that HER3 expression was higher in cell lines with an epithelial phenotype than in those with a mesenchymal phenotype [29, 30], suggesting that HER3 is rather a marker of epithelial traits in HCC.

Autocrine expression of heregulin-1ß has been reported to be a predictive biomarker for response to anti-HER3 antibodies, even in tumours showing no significant prognostic association between heregulin-1ß and OS or PFS [31, 32]. In our HCC collection, the expression of heregulin-1ß transcript was lower in tumours (82 %) than in adjacent tissue, which does not support the existence of heregulin-1ß/HER3 autocrine loops in HCC. Moreover, we have not been able to assign a prognostic value to HER3 and heregulin-1ß mRNA levels. These data contrast with the study by Hsieh and colleagues [20], which reported that upregulation of HER3 mRNA was predictive of early recurrence and poor clinical outcome in a Taiwanese collection of 71 HCC. The reason for such a discrepancy remains unclear. One potential explanation is that the two patient populations diverge in terms of liver-disease etiology. Altogether these data suggest that HCC patients may not derive significant clinical benefit from HER3-directed monotherapies.

There is no standardized method for HER3 detection by immunohistochemistry and information regarding HER3 staining in HCC are limited. The sole extensive study was published with the clone 2F12, which showed frequent HER3 cytoplasmic staining in HCC [18]. In our hands, this clone (and two other ones) did not give signals while the clone RTJ1 yielded cytoplasmic staining but was not reproducible in terms of intensity. The clone RTJ1 has been previously used to detect HER3 in breast [8, 33] and lung [34] cancers. However, the reliability of this clone has been challenged by others [35]. An internationally accepted and validated method for immunohistochemistry detection of HER3 needs consideration.

HER3 is a receptor that is finely regulated at the post-transcriptional and post-translational levels in several cell types. Notably, the steady state level of HER3 protein can be regulated by the ubiquitin-proteasome pathway [25]. Since we did not observe correlation between HER3 mRNA and protein levels in human liver tissue, it is highly probable that HER3 is submitted to post-transcriptional and/or post-translational regulation in this tissue. Although insulin is generally considered as an anabolic hormone that supports protein synthesis and inhibits protein degradation, prolonged exposure of cells to insulin also promotes ubiquitin-proteasome degradation of specific proteins such as insulin-receptor substrate-1 [36] and Foxo1 [37]. Liver tumours express IR and insulinemia plays a key role in the pathogenesis of HCC [38]. We show here that insulin promotes the degradation of HER3 protein in untransformed human hepatocytes and that this negative regulation is maintained in some HCC cell lines such as HepG2 and PLC/PRF5 cells. The pathway whereby insulin acts to repress HER3 protein expression involves proteasome engagement. Further studies are needed to examine whether insulin enhances HER3 ubiquitination in HCC cells. In any case, we did not find evidence of a contributory role of NRDP1 in the effect of insulin.

In HCC cells in which insulin did not promote HER3 degradation, we demonstrate that insulin is able to counteract the pro-migratory effect of heregulin-1ß namely Hep3B and Huh7 cells. The functional interplay between IR and HER3 signalling can be at least partly explained by the physical proximity between the two receptors proved by a coimmunoprecipitation assay. We demonstrate that insulin, through its receptor, was able to rapidly phosphorylate HER3. However, the striking observation was that the insulin-induced HER3 phosphorylation was partial, involving Y1289 but not Y1197 residue. Moreover, we report that the depletion of IR protein with siRNA was accompanied by an increase in heregulin-1ß-induced AKT phosphorylation, indicating that HER3 activation was dependent on IR expression levels. The inhibitory effect of IR on heregulin-1ß/HER3 signalling probably explains the ability of insulin to restrict cell migration in response to heregulin-1ß. The underlying mechanisms remain to be deciphered. In particular, it will be of interest to examine the impact of insulin-induced HER3 phosphorylation on heregulin-1ß affinity and heterodimers balance (EGFR/HER3, IR/HER3) at the plasma membrane.

## Conclusions

Our study highlights several factors (hepatitis virus, insulinemia) that may regulate HER3 expression in HCC and explain why HER3 expression has no prognostic value in a HCC population, which is heterogeneous in terms of etiology. Further studies are required to bring conclusive answers about the clinical significance of HER3 downregulation and inhibition in the setting of insulin signalling.

## Abbreviations

CK19, cytokeratin 19; DAPI, 4′,6-diamidino-2-phenylindole; FBS, fetal bovine serum; HBV, hepatitis B virus; HCC, hepatocellular carcinoma; IR, insulin receptor; NT, non tumour; OS, overall survival; RFS, recurrence-free disease; T, tumour

## Additional files

Additional file 1:
**Table S1.** Antibodies for Western blot and immunofluorescence. Table S2 Primers used for real-time PCR. (DOCX 78 kb) Additional file 2: Figure S1. Evaluation of RTJ1 antibody specificity by Western blot. PLC/PRF5 and HepG2 cells were transiently transfected with a control (siCont) or a siRNA directed against HER3 (siHER3). Whole-cell lysates (20 μg) were analysed by Western blot for HER3 expression using RTJ1 and 2F12 antibodies. (TIF 338 kb) Additional file 3: Figure S2. Prognostic value of heregulin-1ß. Kaplan-Meier analysis of the probabilities of overall survival (*left*) and recurrence-free survival (*right*) according to the upregulation of heregulin-1ß (HRG-1ß) mRNA. Statistical analysis: log-rank test. (TIF 305 kb) Additional file 4: Figure S3. Effect of insulin on HER3 and NDPR1 expression. **A.** HepG2 and PLC/PRF5 cells were treated for 24 h with 10^−8^ M insulin or IGF-II and analysed for HER3 expression by RT-qPCR. **B.** HepG2 and PLC/PRF5 cells were treated for 6, 17 and 48 h with 10^−8^ M insulin and analysed for NRDP1 expression by RT-qPCR (*left*). Cells treated for 48 h with insulin were also analysed for NRDP1 protein expression by Western blot. **C.** PLC/PRF5 cells were transiently transfected with a control (siCont) or siRNA directed against NRDP1 (siNRDP1) and then treated with or without 10^−8^ M insulin for 10 min. Whole-cell lysates (20 μg) were analysed by Western blot for HER3 and NRDP1 expression. β-actin detection was performed to control protein loading. Values are means ± SEM of three independent experiments. Blots are representative of two independent experiments. (TIF 489 kb) Additional file 5: Figure S4. Effect of heregulin-1ß on HCC cell proliferation and viability. **A.** Serum-deprived Hep3B and Huh7 cells were treated with 0.3 % FBS, 10 % FBS or 0.3 % FBS plus heregulin-1ß (HRG-1ß, 50 ng/µl) and cell number were counted at day 1, 2 and 3. **B.** Serum-deprived Hep3B and Huh7 cells were treated with 0.3 % FBS, 10 % FBS or 0.3 % FBS plus heregulin-1ß (50 ng/µl) and cell viability was determined using a MTT assay. (TIF 332 kb) Additional file 6: Figure S5. Effects of insulin on HER3 phosphorylation in HCC cell lines. Huh7, HepG2 and PLC/PRF5 cells were treated with insulin (*left:* increasing doses during 10 min; *right:* 10^−8^ M for different times). Whole-cell lysates (20 μg) were analysed by Western blot for phosphorylation and expression of HER3 and/or IR. (TIFF 1.16 mb)
