# Erratum to: Heregulin-1ß and HER3 in hepatocellular carcinoma: status and regulation by insulin

**DOI:** 10.1186/s13046-016-0438-4

**Published:** 2016-09-28

**Authors:** Corina Buta, Eva Benabou, Marie Lequoy, Hélène Régnault, Dominique Wendum, Fatiha Merabtene, Hamza Chettouh, Lynda Aoudjehane, Filomena Conti, Yves Chrétien, Olivier Scatton, Olivier Rosmorduc, Françoise Praz, Laetitia Fartoux, Christèle Desbois-Mouthon

**Affiliations:** 1 Sorbonne Universités, UPMC Univ Paris 06, INSERM, Saint-Antoine Research Center, 34 rue Crozatier, F-75012 Paris, France; 2 Department of Hepatology, AP-HP, Saint-Antoine Hospital, F-75012 Paris, France; 3 Department of Hepatology, AP-HP, Pitié-Salpétrière Hospital, F-75013 Paris, France; 4 Department of Pathology, AP-HP, Saint-Antoine Hospital, F-75012 Paris, France; 5 Histomorphology Platform, UMS 30 Lumic, F-75012 Paris, France; 6 Human HepCell, Saint-Antoine Hospital, F-75012 Paris, France; 7 Department of Hepatobiliary Surgery and Liver Transplantation, AP-HP, Pitié-Salpétrière Hospital, F-75013 Paris, France

## Erratum description

In the original publication of this article [1], one of the co-authors’ names contained a spelling error. ‘Fatiha Meratbene’ should therefore have been listed as ‘Fatiha Merabtene’. The original publication has now been modified as well to reflect these changes.
