# Supplementary material for: Heregulin-1ß and HER3 in hepatocellular carcinoma: status and regulation by insulin
Source: J Exp Clin Cancer Res. 2016 Aug 11;35:126. doi: 10.1186/s13046-016-0402-3 (PMC4982118; doi:10.1186/s13046-016-0402-3)
Supplement: Additional file 1: — Table S1. Antibodies for Western blot and immunofluorescence. Table S2 Primers used for real-time PCR. (DOCX 78 kb) [file 13046_2016_402_MOESM1_ESM.docx]

***Table S1: Antibodies for Western blot and immunofluorescence***

| **Antibody** | **Reference** | **Provider** | **Application** |
| --- | --- | --- | --- |
| pIR (19H7) | 3024 | Cell Signaling Technology, Inc. | WB |
| IR (C-19) | sc-711 | Santa Cruz Biotechnology, Inc. | WB |
| IR | Ab 5500 | Abcam | IF |
| pAKT (S473) (D9E) | 4060 | Cell Signaling Technology, Inc. | WB |
| AKT (C67E7) | 4691 | Cell Signaling Technology, Inc. | WB |
| pHER3 (Y1289) (21D3) | 4791 | Cell Signaling Technology, Inc. | WB |
| pHER3 (Y1197 (C56E4) | 4561 | Cell Signaling Technology, Inc. | WB |
| HER3 (1B2E) | 4754 | Cell Signaling Technology, Inc. | WB |
| HER3 (Ab-4) | OP119 | Merck Millipore | IF |
| HER3 (RTJ2) | sc-415 | Santa Cruz Biotechnology, Inc. | IHC |
| HER3 (RTJ1) | MA1-22819 | Thermo scientific | IHC |
| HER3 (DAK-H3-IC) | M729729 | DAKO | IHC |
| HER3 (2F12) | 05-390 | Merck Millipore | IHC |
| EGFR (1005) | sc-03 | Santa Cruz Biotechnology, Inc. | WB |
| NRDP1 (B-8) | sc-374120 | Santa Cruz Biotechnology, Inc. | WB |
| β-actin (AC-15) | A5441 | Sigma Aldrich | WB |

IF, immunofluorescence ; IHC, immunohistochemistry ; WB, Western blot

***Table S2: Primers used for real-time PCR***

| **Target** | **Forward primer** | **Reverse primer** | **Product size (bp)** |
| --- | --- | --- | --- |
| **HER3** | CTGATCACCGGCCTCAAT | GGAAGACATTGAGCTTCTCTGG | 72 |
| **Heregulin-1β** | TGGCTGACAGCAGGACTAAC | CTGGCCTGGATTTCTTC | 60 |
| **NRDP1** | tgaatacaacgagatcctagagtgg | cacagcatcaggagtcgaga | 90 |
| **HPRT** | TAATTGGTGGAGATGATCT | TGCCTGACCAAGGAAAGC | 108 |
