# Supplementary figures and images for: Heregulin-1ß and HER3 in hepatocellular carcinoma: status and regulation by insulin
Source: J Exp Clin Cancer Res. 2016 Aug 11;35:126. doi: 10.1186/s13046-016-0402-3 (PMC4982118; doi:10.1186/s13046-016-0402-3)

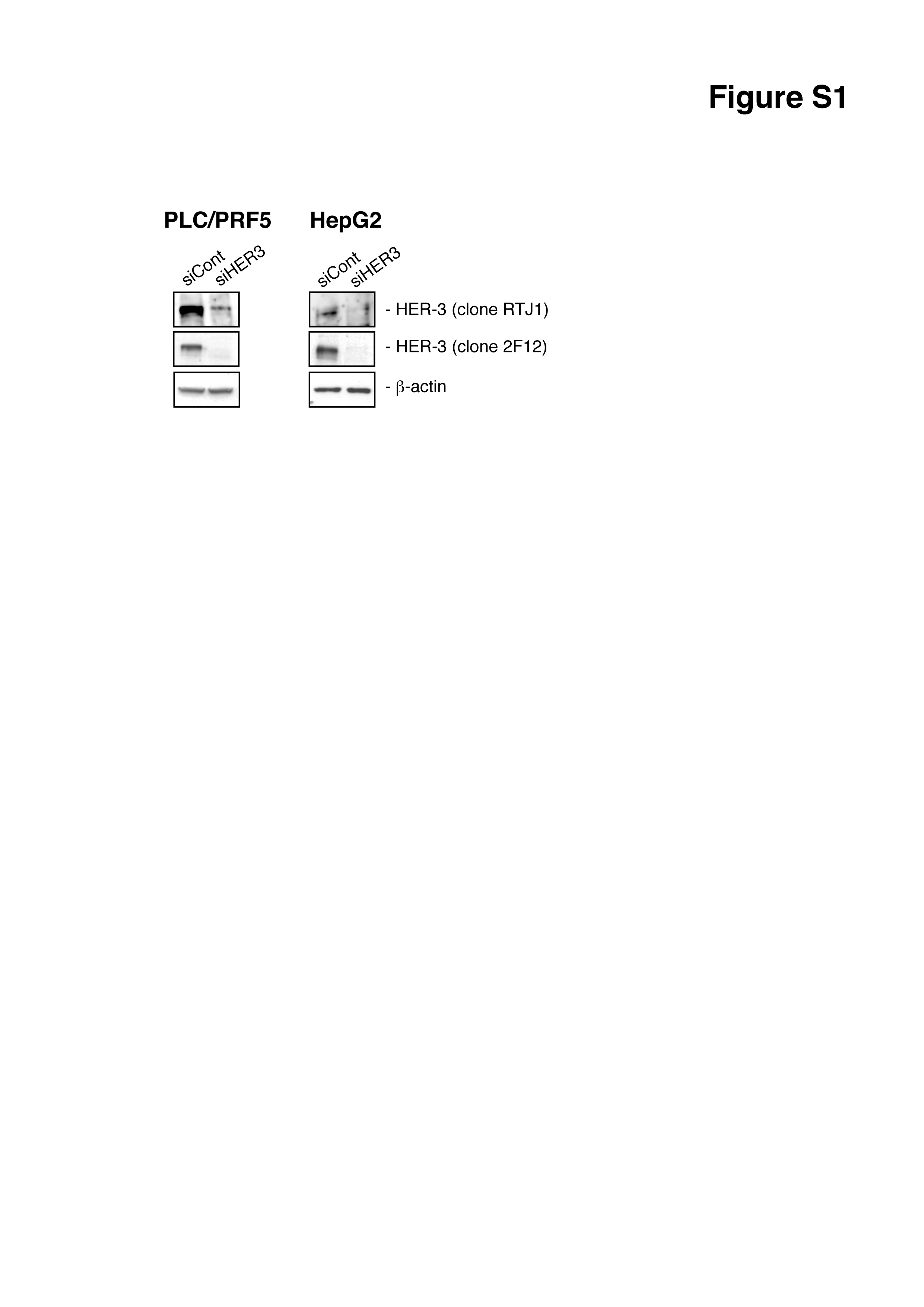

Supplement: Additional file 2: Figure S1. — Evaluation of RTJ1 antibody specificity by Western blot. PLC/PRF5 and HepG2 cells were transiently transfected with a control (siCont) or a siRNA directed against HER3 (siHER3). Whole-cell lysates (20 μg) were analysed by Western blot for HER3 expression using RTJ1 and 2F12 antibodies. (TIF 338 kb) [file 13046_2016_402_MOESM2_ESM.tif]

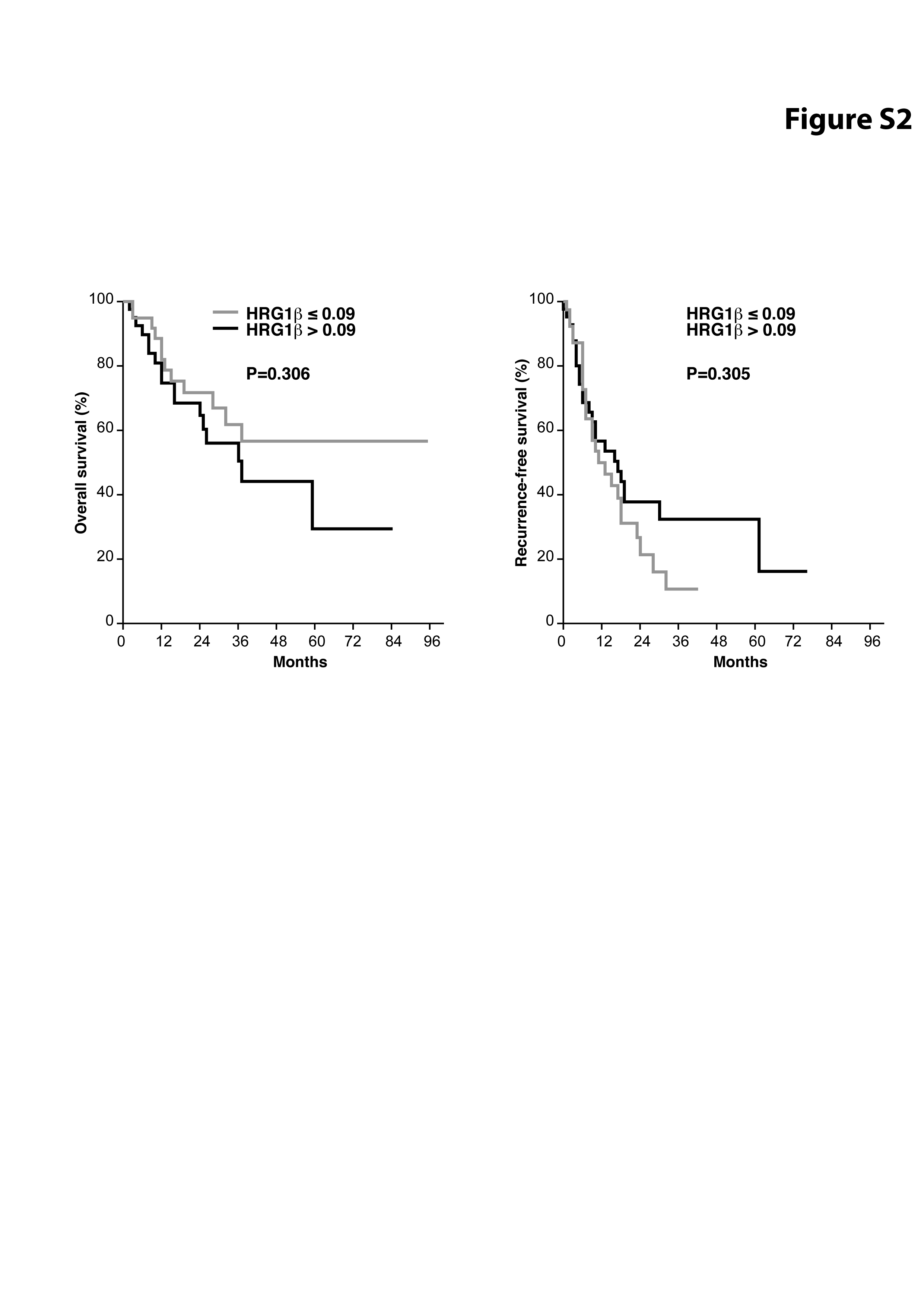

Supplement: Additional file 3: Figure S2. — Prognostic value of heregulin-1ß. Kaplan-Meier analysis of the probabilities of overall survival (left) and recurrence-free survival (right) according to the upregulation of heregulin-1ß (HRG-1ß) mRNA. Statistical analysis: log-rank test. (TIF 305 kb) [file 13046_2016_402_MOESM3_ESM.tif]

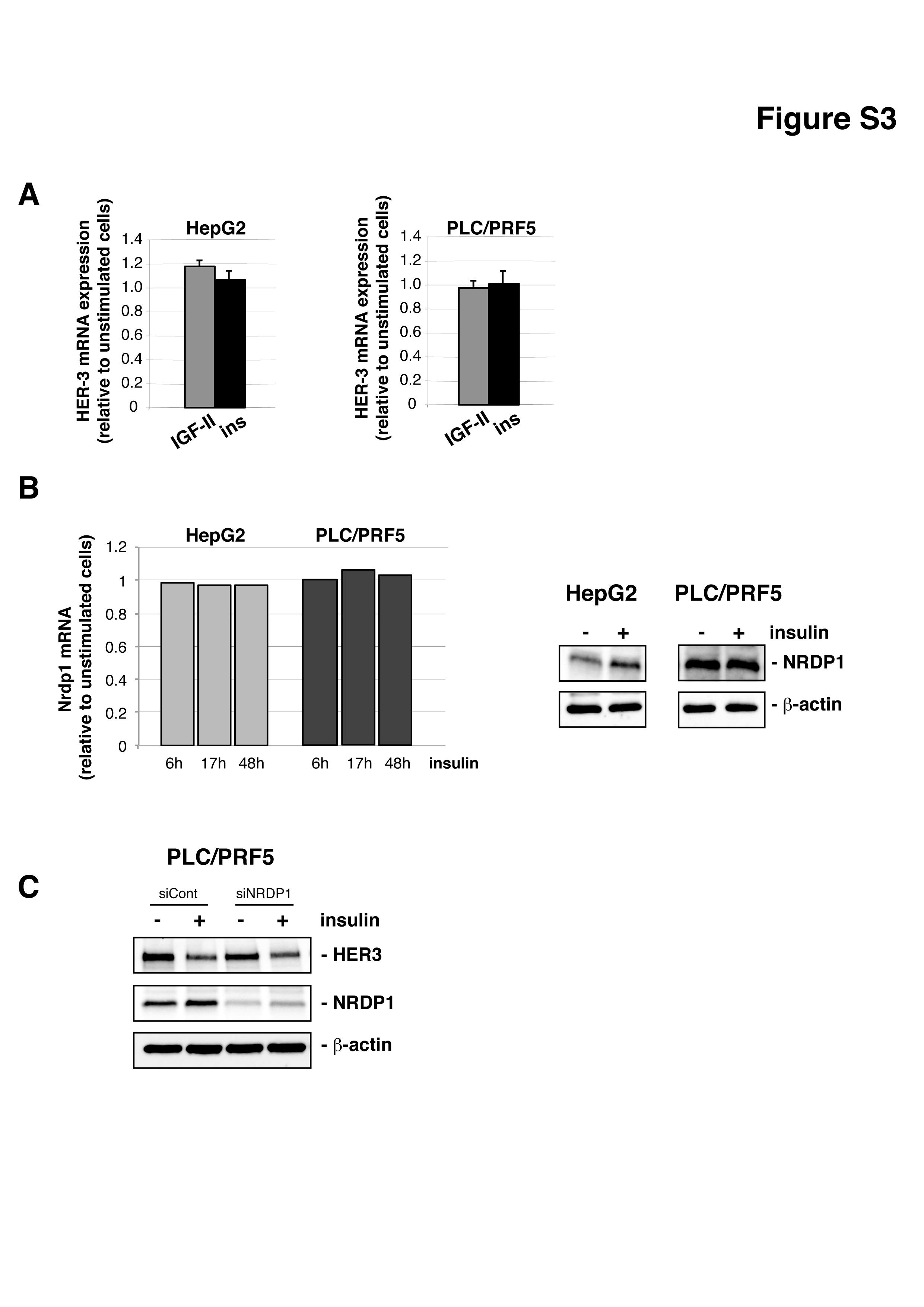

Supplement: Additional file 4: Figure S3. — Effect of insulin on HER3 and NDPR1 expression. A. HepG2 and PLC/PRF5 cells were treated for 24 h with 10−8 M insulin or IGF-II and analysed for HER3 expression by RT-qPCR. B. HepG2 and PLC/PRF5 cells were treated for 6, 17 and 48 h with 10−8 M insulin and analysed for NRDP1 expression by RT-qPCR (left). Cells treated for 48 h with insulin were also analysed for NRDP1 protein expression by Western blot. C. PLC/PRF5 cells were transiently transfected with a control (siCont) or siRNA directed against NRDP1 (siNRDP1) and then treated with or without 10−8 M insulin for 10 min. Whole-cell lysates (20 μg) were analysed by Western blot for HER3 and NRDP1 expression. β-actin detection was performed to control protein loading. Values are means ± SEM of three independent experiments. Blots are representative of two independent experiments. (TIF 489 kb) [file 13046_2016_402_MOESM4_ESM.tif]

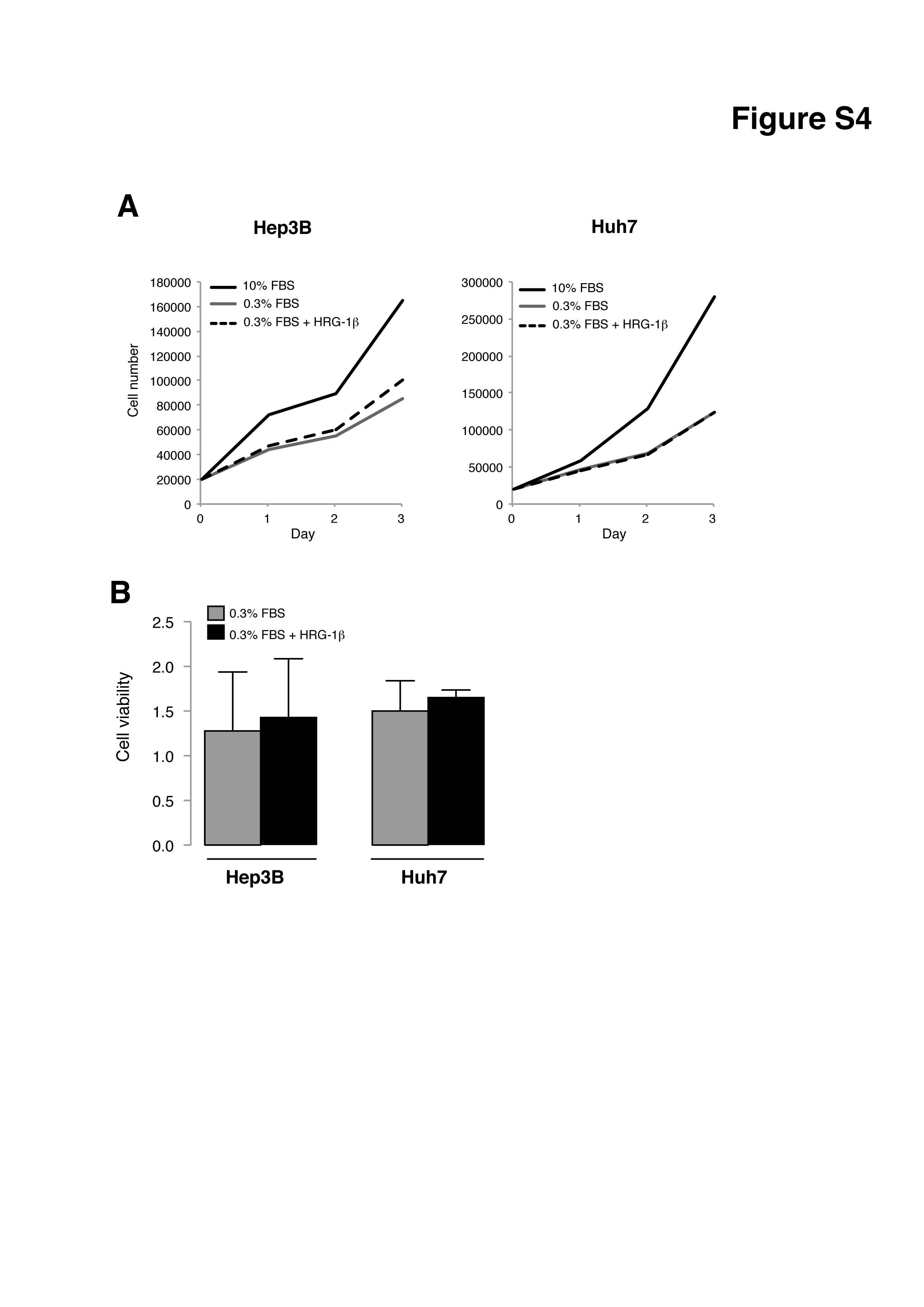

Supplement: Additional file 5: Figure S4. — Effect of heregulin-1ß on HCC cell proliferation and viability. A. Serum-deprived Hep3B and Huh7 cells were treated with 0.3 % FBS, 10 % FBS or 0.3 % FBS plus heregulin-1ß (HRG-1ß, 50 ng/µl) and cell number were counted at day 1, 2 and 3. B. Serum-deprived Hep3B and Huh7 cells were treated with 0.3 % FBS, 10 % FBS or 0.3 % FBS plus heregulin-1ß (50 ng/µl) and cell viability was determined using a MTT assay. (TIF 332 kb) [file 13046_2016_402_MOESM5_ESM.tif]

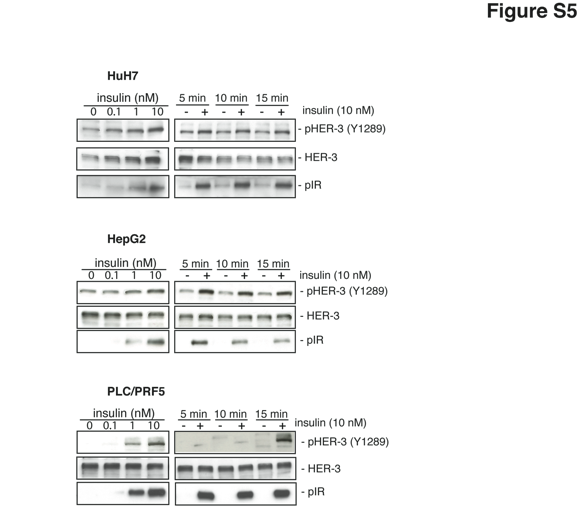

Supplement: Additional file 6: Figure S5. — Effects of insulin on HER3 phosphorylation in HCC cell lines. Huh7, HepG2 and PLC/PRF5 cells were treated with insulin (left: increasing doses during 10 min; right: 10−8 M for different times). Whole-cell lysates (20 μg) were analysed by Western blot for phosphorylation and expression of HER3 and/or IR. (TIFF 1.16 mb) [file 13046_2016_402_MOESM6_ESM.tiff]
